# Supplementary figures and images for: A unique GCN5 histone acetyltransferase complex controls erythrocyte invasion and virulence in the malaria parasite Plasmodium falciparum
Source: PLoS Pathog. 2021 Aug 17;17(8):e1009351. doi: 10.1371/journal.ppat.1009351 (PMC8396726; doi:10.1371/journal.ppat.1009351)

S1 Fig

A.

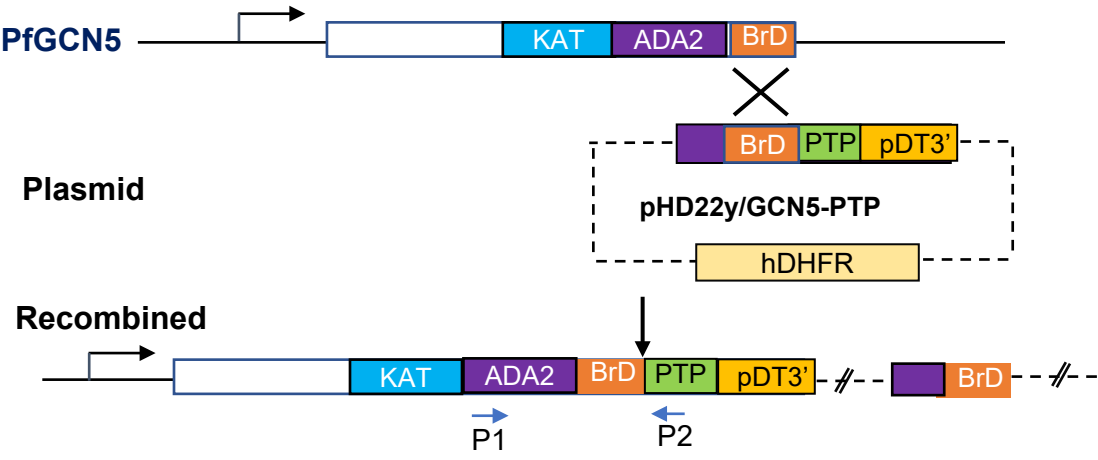

B.

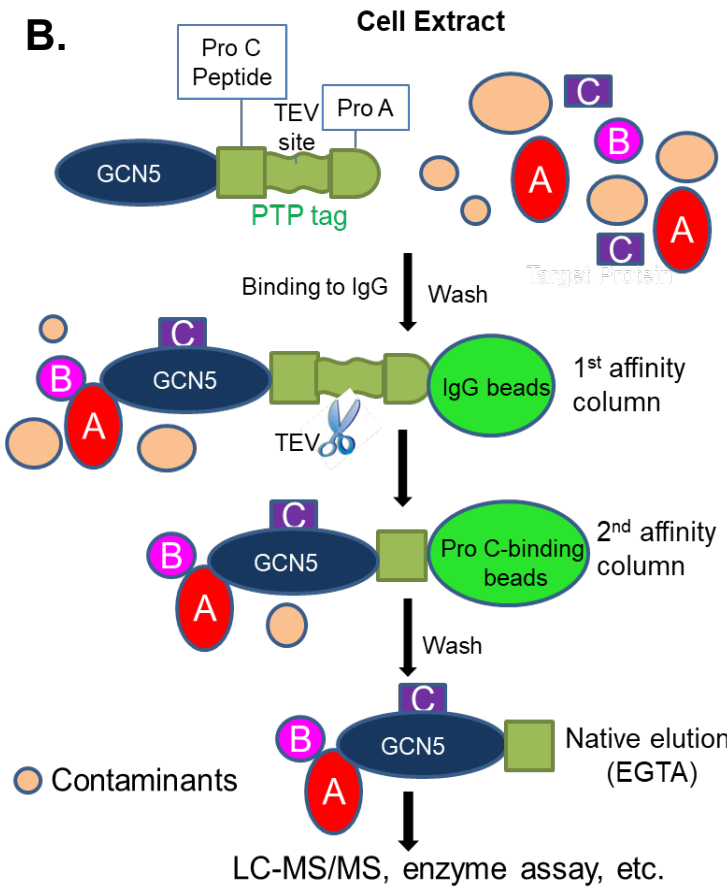

C.

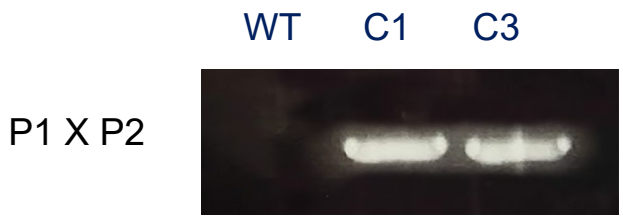

E.

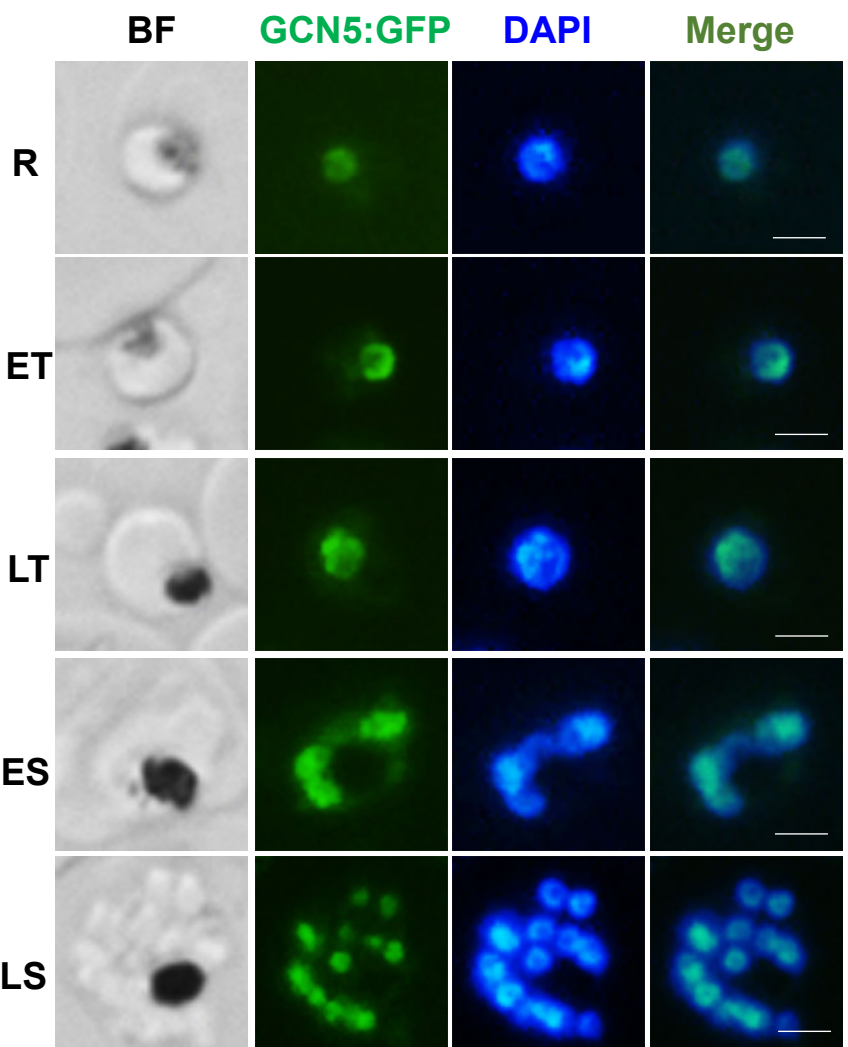

D.

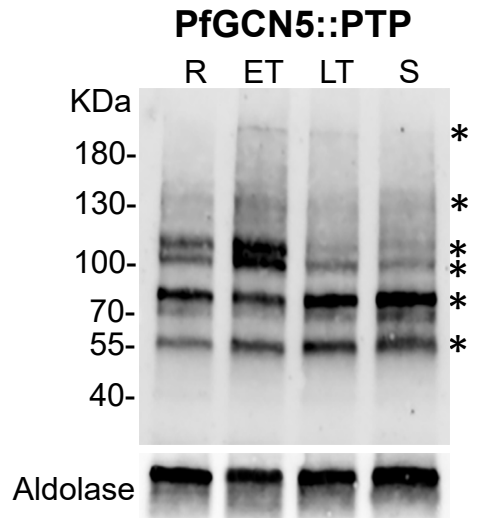

Supplement: S1 Fig — (A) Schematic diagram of PTP tagging at C-terminal of PfGCN5. P1 and P2 are primers used for verification of integration by PCR. (B) Cartoon shows the TAP procedure for purification of the PfGCN5 complex. A, B, and C are subunits of the GCN5 complex. TEV: tobacco etch virus protease. (C) Integration-specific PCR verification of two positive clones (C1 and C3) from transfected parasites. (D) Western blot detecting PfGCN5::PTP expression in the recombinant parasite clone C3 at different developmental stages (R: ring; ET: early trophozoite; LT: late trophozoite; S: schizont). The blot was probed with antibodies against protein C. Molecular markers in kDa are shown on the left. The expression of aldolase was used as a loading control. The PfGCN5::PTP protein bands are indicated by asterisks. (E) Live cell imaging shows the localization of PfGCN5::GFP during intraerythrocytic development (R: ring; ET: early trophozoite; LT: late trophozoite; ES: early schizont; LS: late schizont). DAPI was used to stain nucleus. BF, bright field. Scale bar = 5 μm. (PDF) [file ppat.1009351.s001.pdf]

S3 Fig

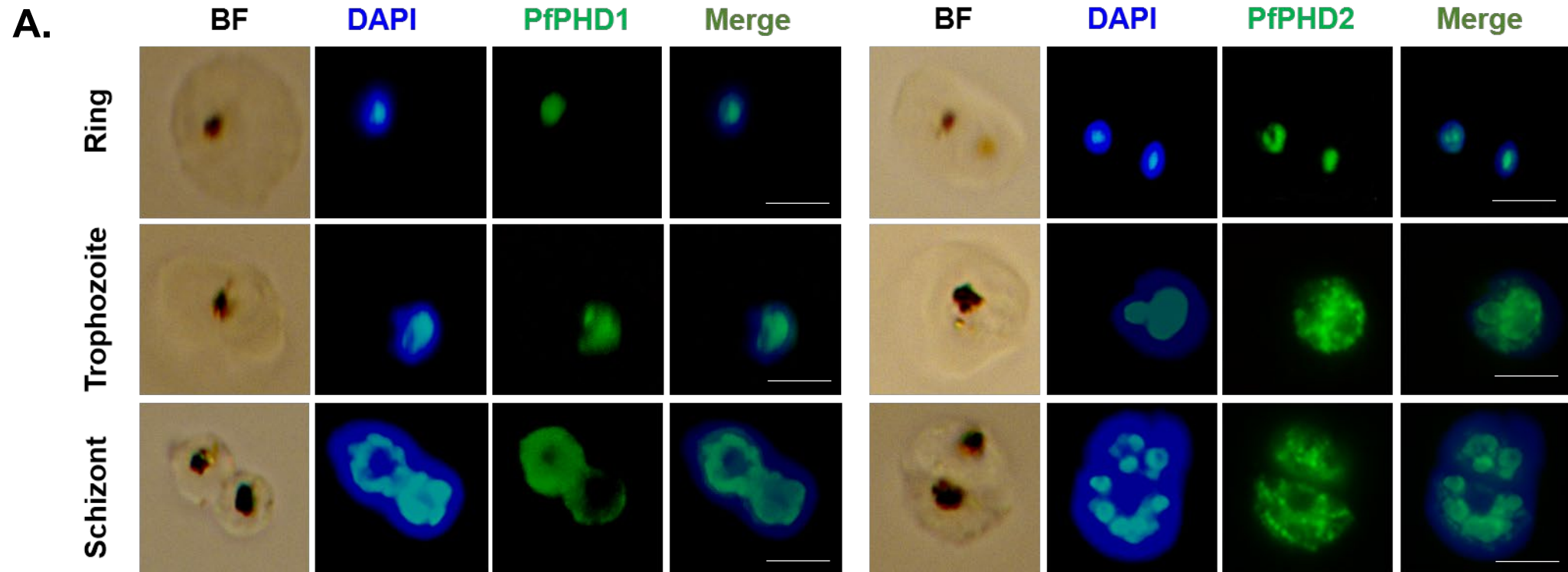

**B.**

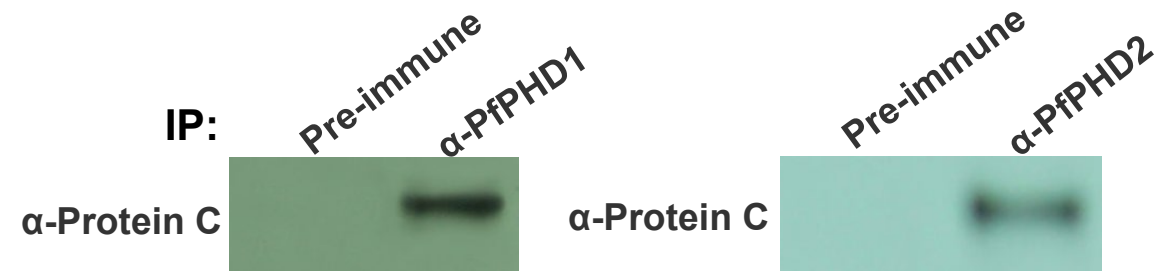

**C.**

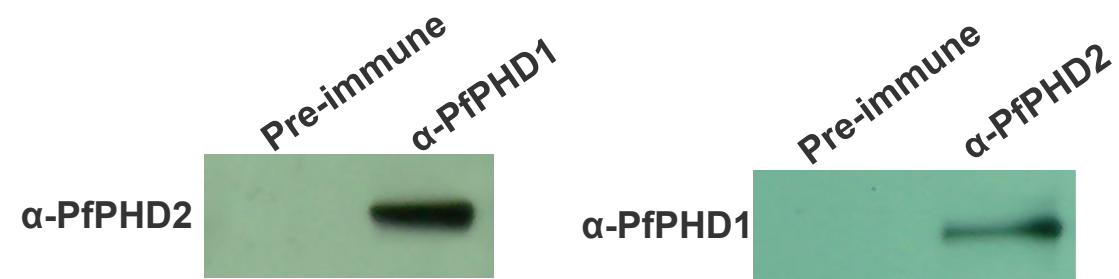

Supplement: S3 Fig — (A) The nuclear localization of PfPHD1 and PfPHD2 were interrogated by IFA using anti-PfPHD1 and PfPHD2 antibodies. Nuclei were counter-stained by DAPI. Scale bar = 5 μm. BF, bright field. (B) Immunoprecipitation (IP) of proteins from lysates of the PfGCN5::PTP parasite line using agarose conjugated with either anti-PfPHD1 or PfPHD2 antibodies. IPed proteins were separated by SDS-PAGE and probed with anti-Protein C antibodies recognizing the full-length GCN5::PTP. Pre-immune sera were used as controls. (C) IP of proteins from lysates of the 3D7 wild-type parasites using agarose conjugated with anti-PfPHD1 (left panel) or PfPHD2 (right panel) antibodies. IPed proteins were separated by SDS-PAGE and probed with anti-PfPHD2 or anti-PfPHD1 antibodies. (PDF) [file ppat.1009351.s003.pdf]

S4 Fig

A.

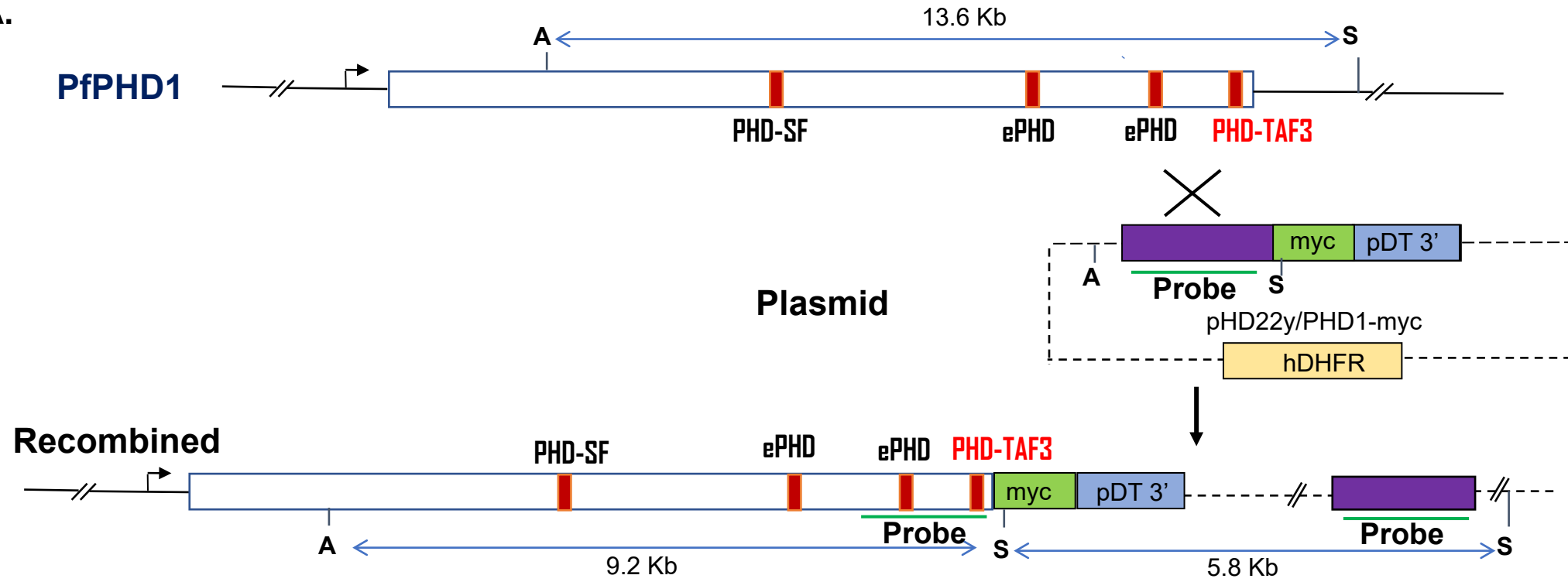

B.

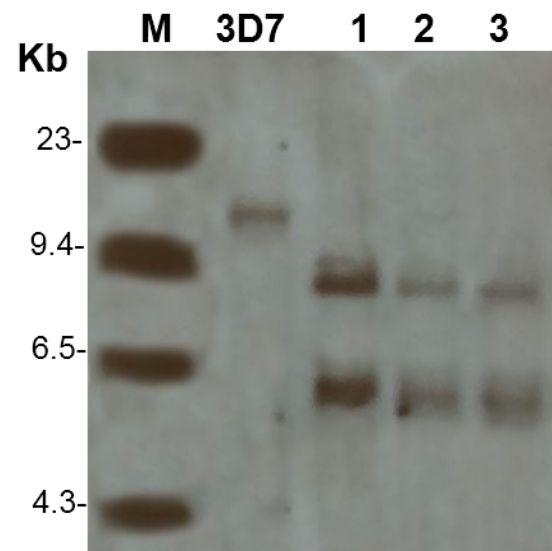

C.

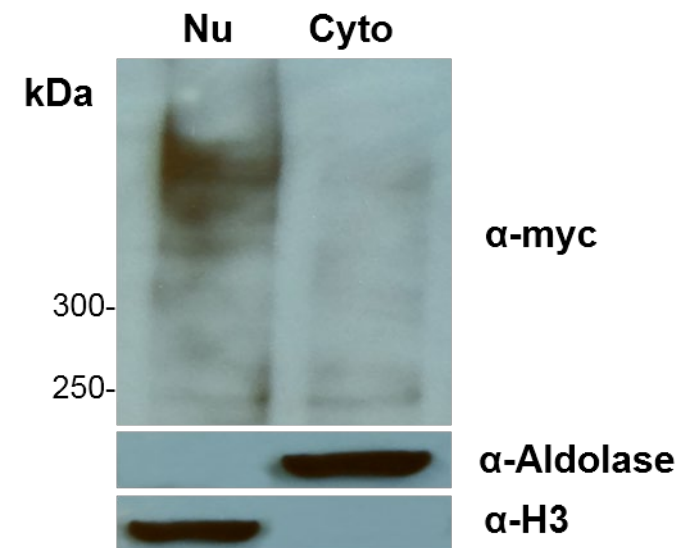

Supplement: S4 Fig — (A) Schematic diagram of Myc tagging at C-terminal of PfPHD1. A, AvrII; S, StuI. (B) Southern blot of 3D7 and three transgenic clones (1–3). Genomic DNA was digested with AvrII and StuI and hybridized with labeled DNA shown as “Probe” in A. (C) Western blot analysis of nuclear (Nu) and cytoplasmic (Cyto) protein extracts with antibodies against the Myc tag, aldolase (for cytoplasmic compartment) and histone H3 (for nuclear compartment). (PDF) [file ppat.1009351.s004.pdf]

S5 Fig

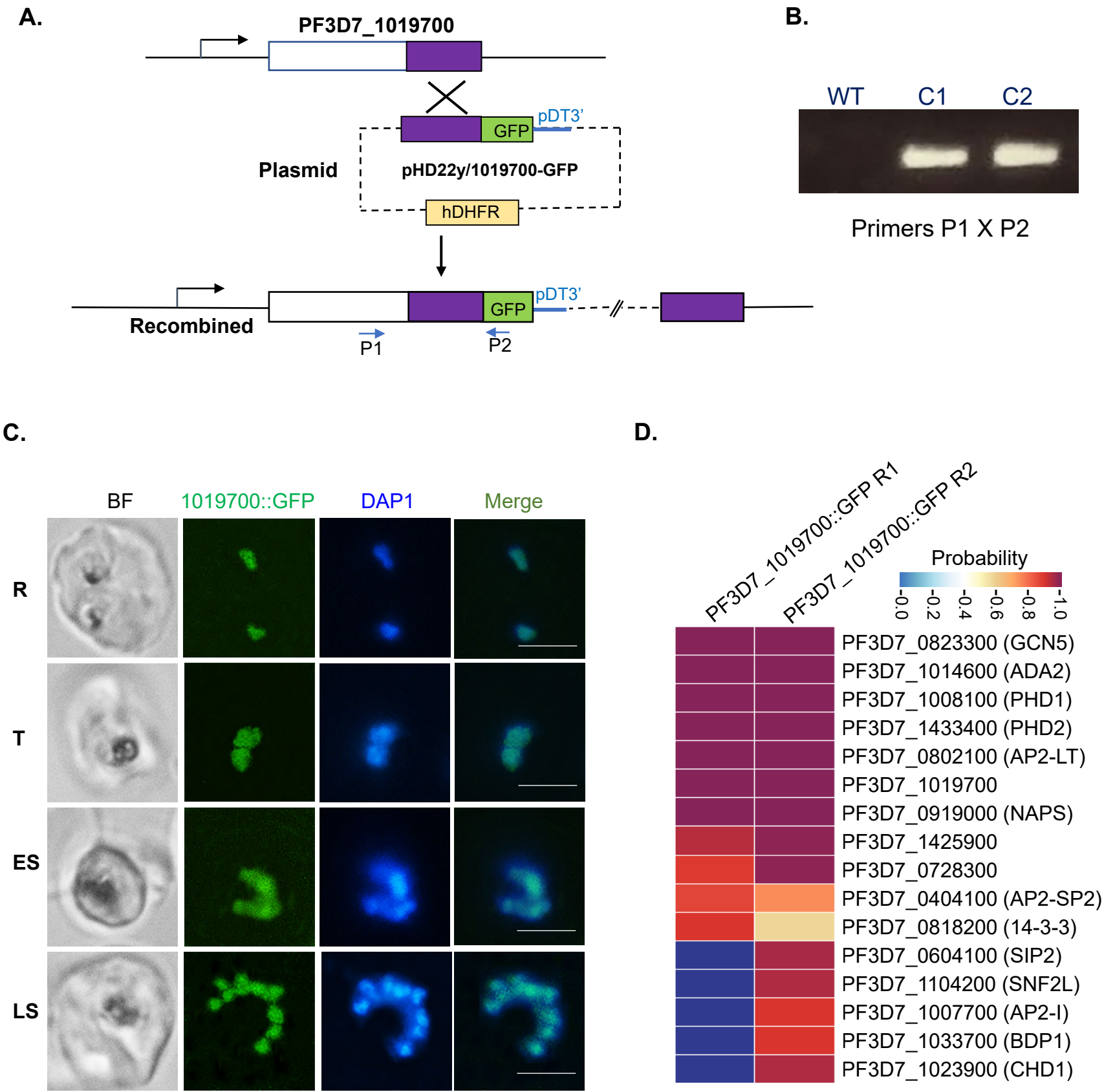

Supplement: S5 Fig — (A) The diagram shows GFP tagging of the PF3D7_1019700 at its C-terminus by single-crossover homologous recombination. Purple blocks show the fragment used for homologous recombination. (B) Integration-specific PCR using primers P1 and P2. WT, Wildtype 3D7; C1 and C2 are two transgenic clones. (C) Live cell imaging shows the localization of PF3D7_1019700::GFP in the nuclei by fluorescence microscopy. R: ring; ET: early trophozoite; LT: late trophozoite; ES: early schizont; LS: late schizont. (D) Proteins identified from parasite nuclear extracts by IP and LC-MS/MS. Single-step IPs with anti-GFP beads were done using the PF3D7_1019700::GFP (R1 and R2 indicate two replicates). The wild-type 3D7 was used as the IP control. The proteomic data were analyzed by SAINT using a threshold of probability >94% and 1% FDR. Gene ID and annotations are shown on the right. (PDF) [file ppat.1009351.s005.pdf]

# S6 Fig

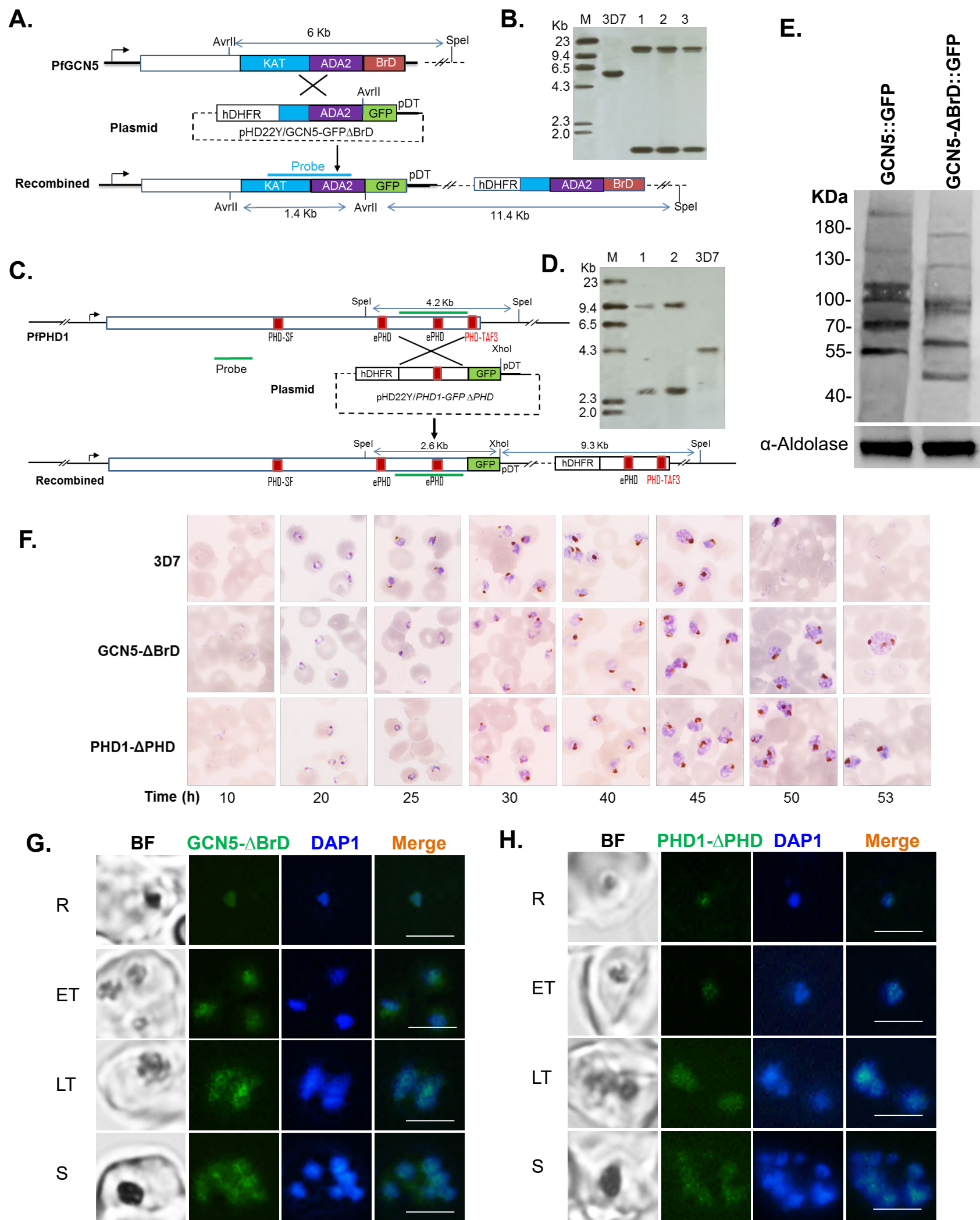

Supplement: S6 Fig — (A) Schematic showing BrD deletion by single crossover homologous recombination. (B) Southern blot analysis of three positive clones from transfected parasites. Genomic DNA was digested with AvrII and SpeI, and hybridized to the probe marked in A. (C) Schematic showing the deletion of PHD-TAF3 domain. (D) Southern blot of two positive clones from transfected parasites. Genomic DNA was digested with SpeI and XhoI, and hybridized to the probe marked in C. In both cases, GFP was tagged at the ends of truncated genes. (E) Western blot shows the size changes of the truncated GCN5 protein bands and the reduced expression levels after BrD deletion. (F). Images of Giemsa-stained films of parasite cultures synchronized at the ring stage to show the extended IDC of the two domain deletion parasite lines. (G) Live cell imaging shows GFP signals in parasites with truncated GCN5 in the GCN5-ΔBrd::GFP parasite line. Compared to S1E Fig, the GCN5-ΔBrd-GFP protein shows weaker fluorescence and a more diffused nuclear localization pattern. Scale bar = 5 μm. (H) Localization of truncated PfPHD1 in PHD1-ΔPHD::GFP parasite line. Scale bar = 5 μm. R, ET, LT and S denote ring, early trophozoite, late trophozoite and schizont stages, respectively. BF, bright field. (PDF) [file ppat.1009351.s006.pdf]

**S7 Fig**

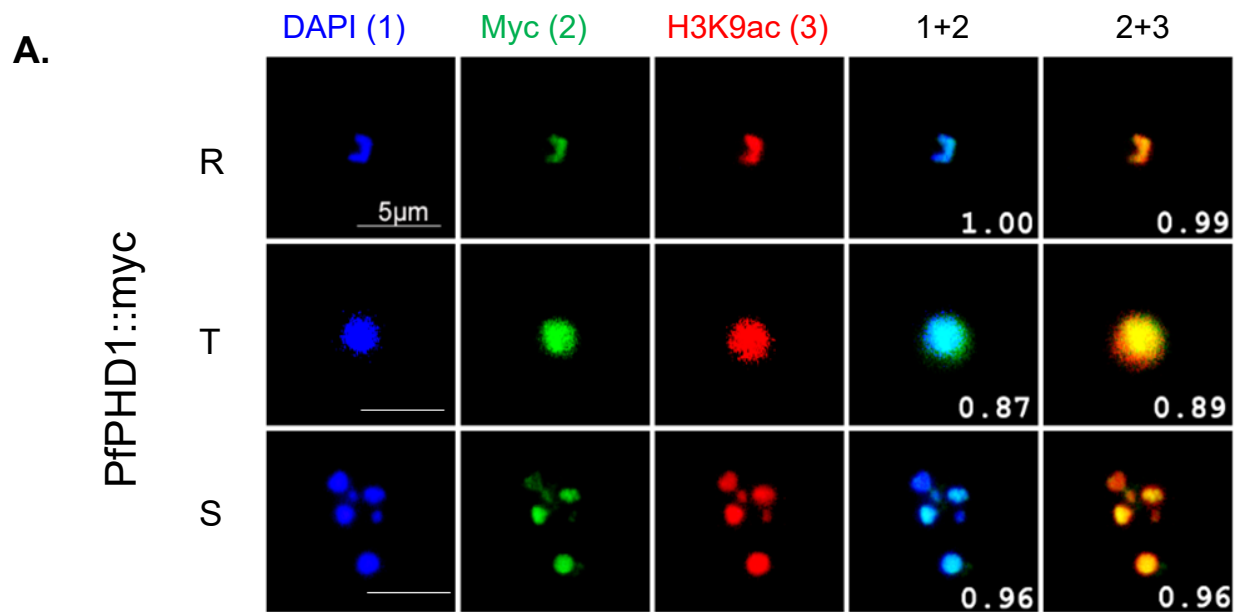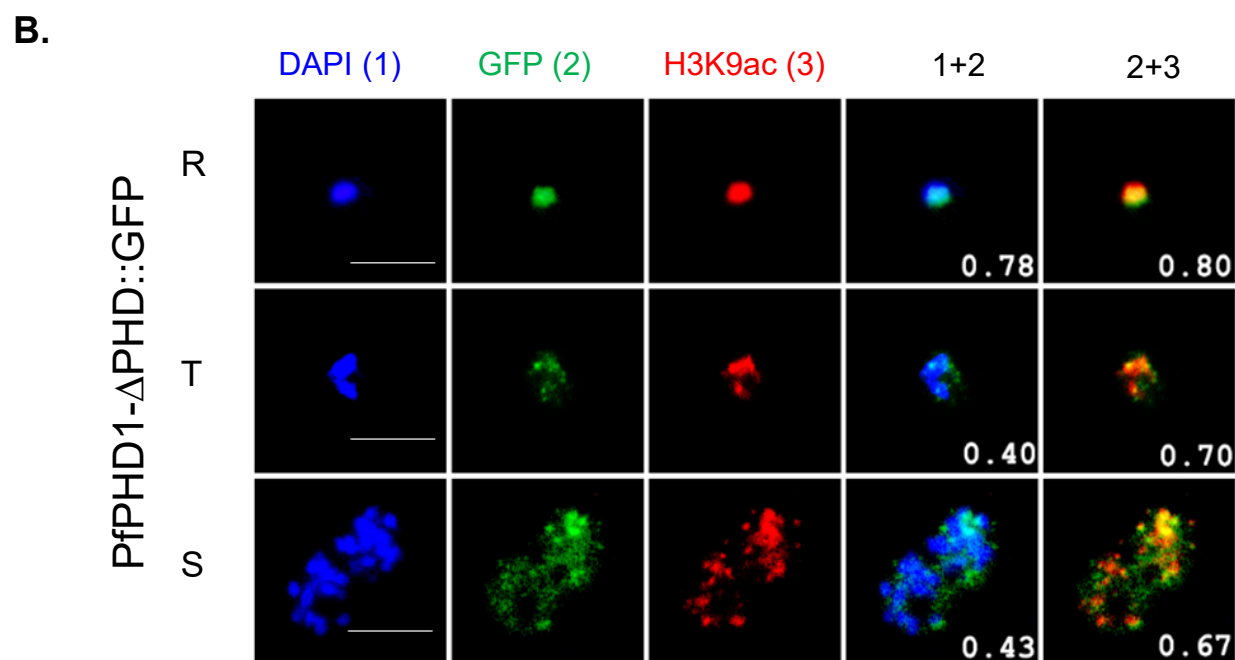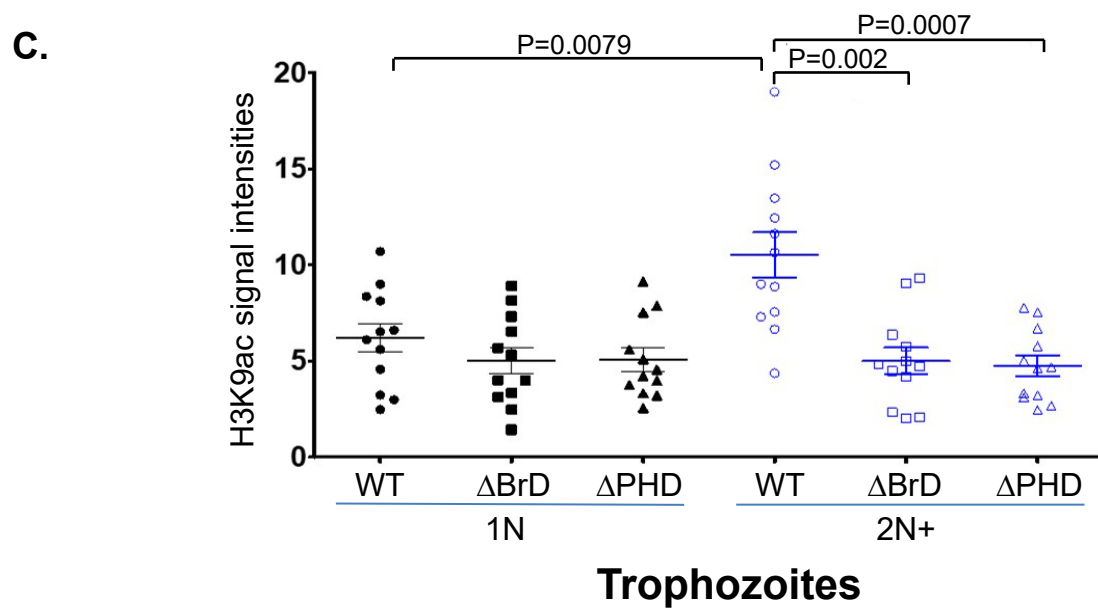

Supplement: S7 Fig — (A) IFA images show high-degree co-localization of PfPHD1, H3K9ac and euchromatin (DAPI) in the nucleus in parasite line with c-Myc-tagged PfPHD1. Numbers indicate the levels of signal correlation between two markers. Scale bar = 5 μm. (B) IFA shows decreased co-localization of PfPHD1, H3K9ac and euchromatin (denoted by DAPI staining) after PHD1 PHD domain deletion. Scale bar = 5 μm. R, ring; T, trophozoite; S, schizont. (C) The intensities of H3K9ac in the 1N or 2N+ single nucleus of wild-type (WT), PfGCN5-ΔBrD::GFP, and PfPHD1-ΔPHD::GFP trophozoite. The copy number (1N or 2N+) in a single nucleus of trophozoite was defined by the intensity of DAPI signal compared to the 1N nucleus of the ring. Bars indicate means and standard deviations (n = 12). The statistical difference was evaluated by Mann–Whitney U test. (PDF) [file ppat.1009351.s007.pdf]

**S8 Fig**

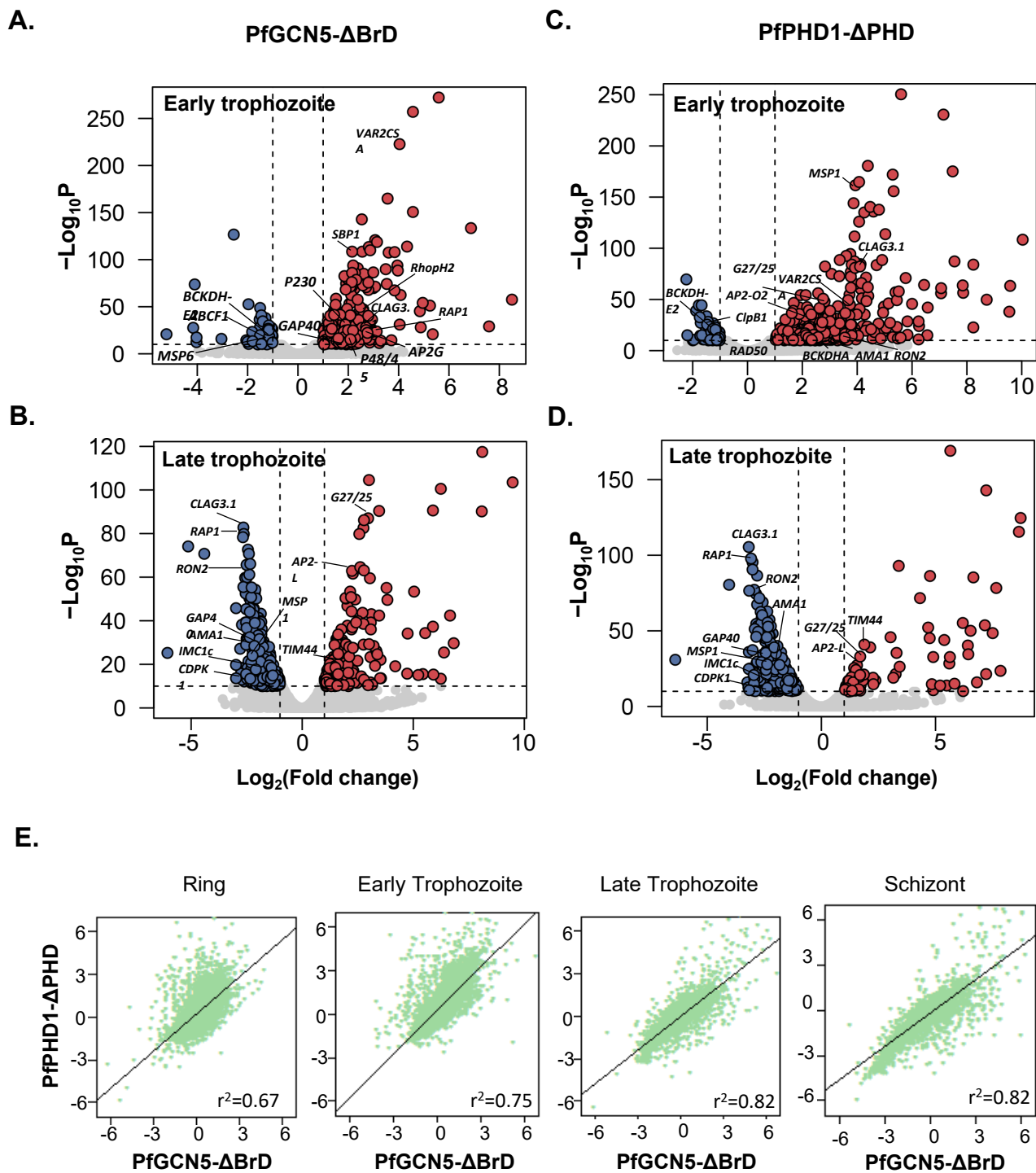

Supplement: S8 Fig — (A–D) Volcano plots show the genes with altered transcription at the early trophozoite (A) and late trophozoite (B) stages in PfGCN5-ΔBrD, and at the early trophozoite (C) and late trophozoite (D) stages in PfPHD1-ΔPHD. (E) Pearson correlation in fold change between PfGCN5-ΔBrD and PfPHD1-ΔPHD in different developmental stages. (PDF) [file ppat.1009351.s008.pdf]

S9 Fig

A.

PfGCN5-ΔBrD

Up-regulated

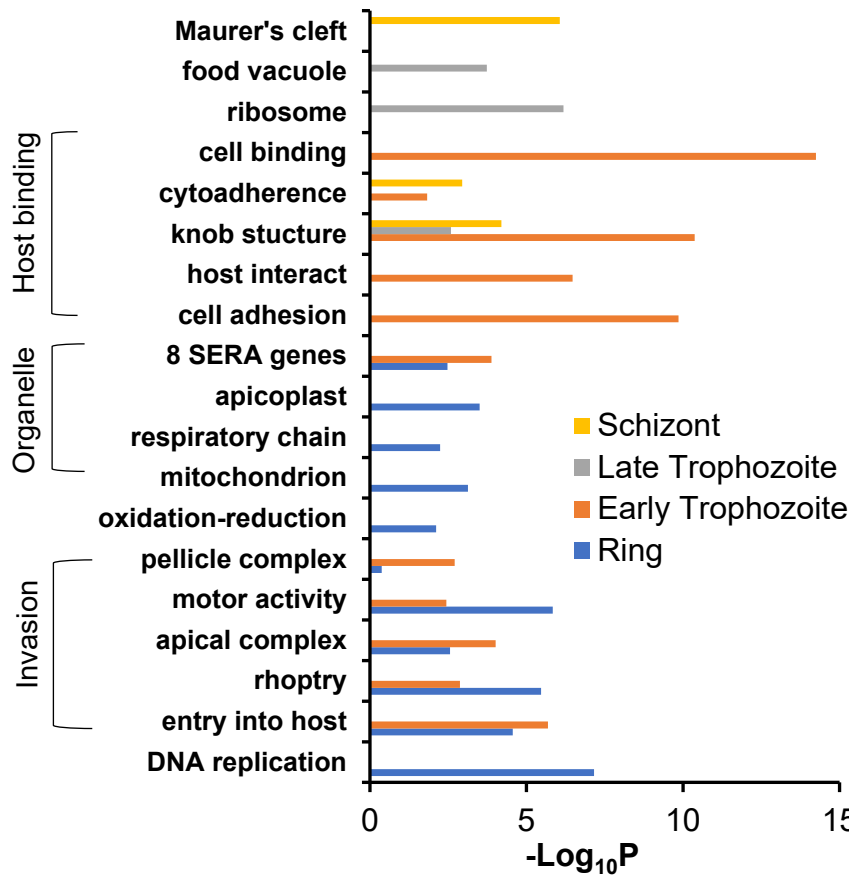

B.

Down-regulated

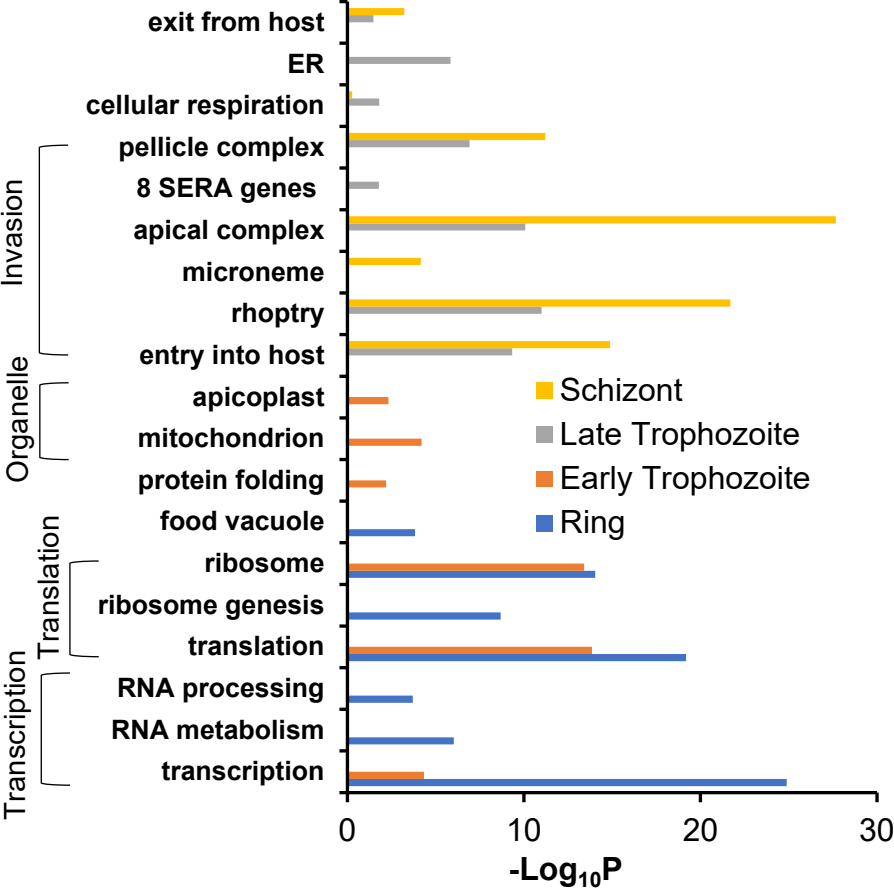

C.

PfPHD1-ΔPHD

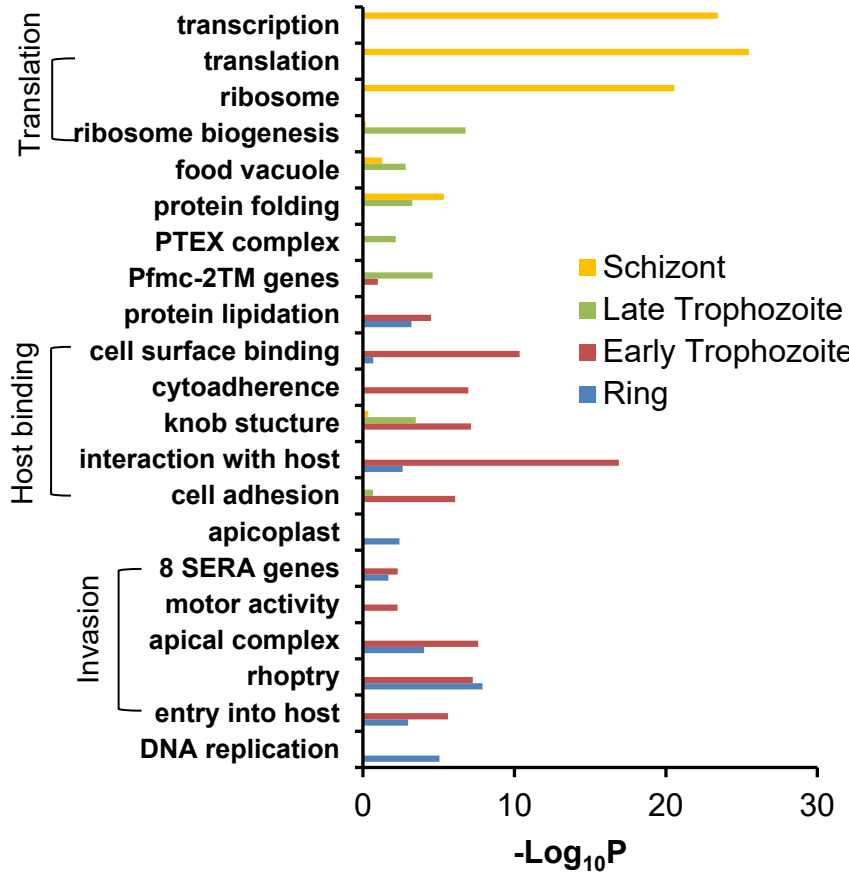

D.

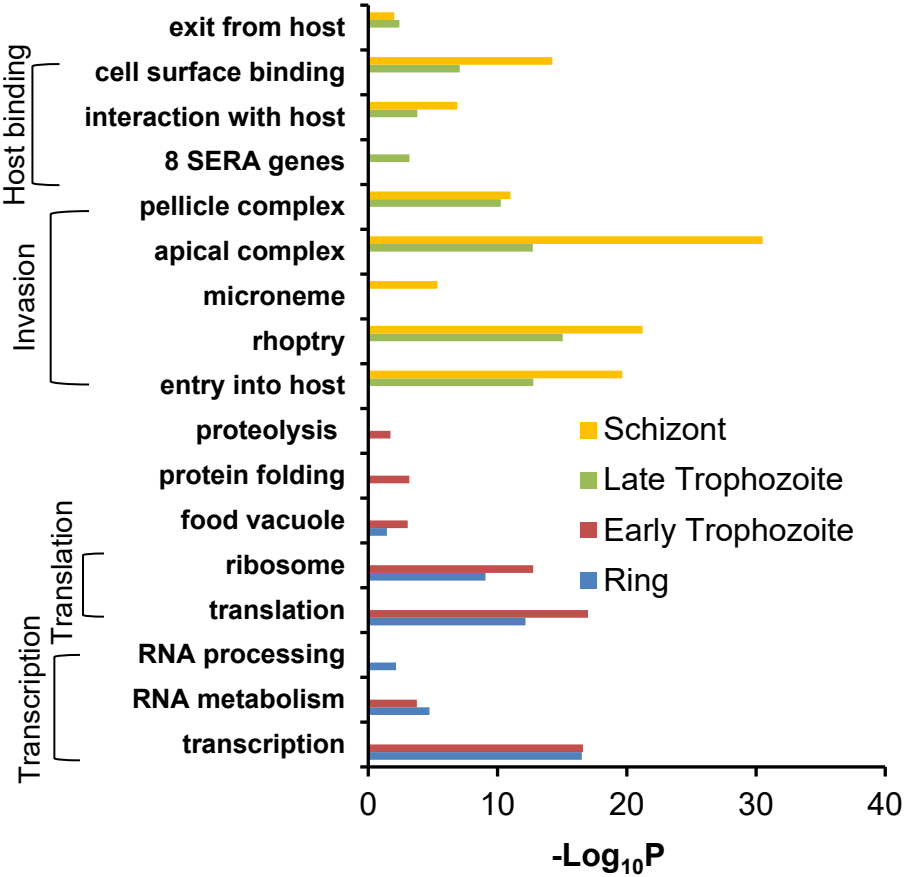

Supplement: S9 Fig — Gene ontology enrichment analysis of up- (A, C) and down-regulated (B, D) genes in PfGCN5-ΔBrD (A, B) and PfPHD1-ΔPHD (C, D) parasites compared to the wildtype 3D7. (PDF) [file ppat.1009351.s009.pdf]

S10 Fig

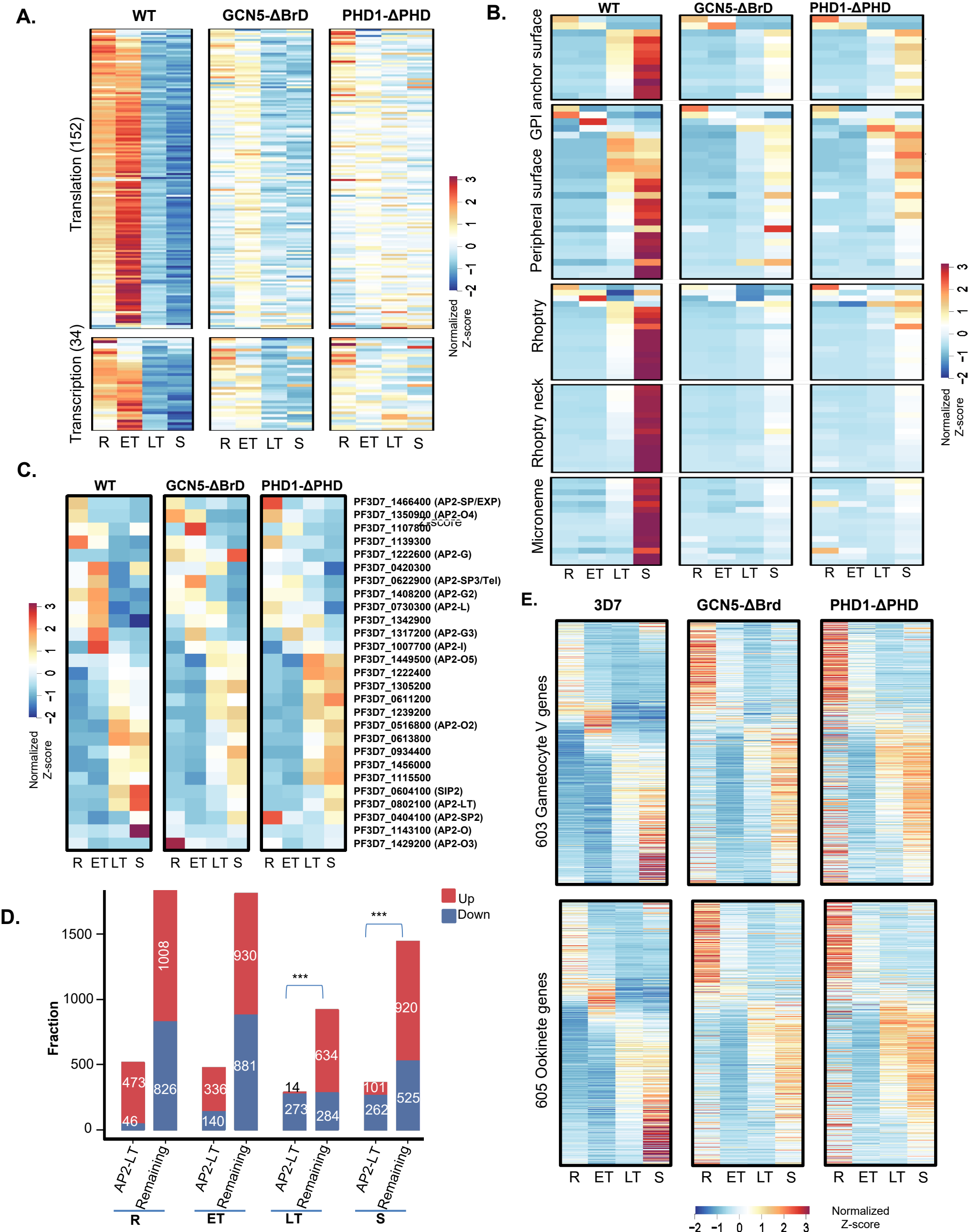

Supplement: S10 Fig — (A-D) Heatmaps display the alteration of gene transcription showing normalized z-scores to illustrate the gene expression cascade observed in 3D7 and those in mutant parasite lines. (A) Genes associated with protein translation and gene transcription. (B) Genes involved in the invasion of RBCs. (C) AP2-family genes. (D) Genes specifically expression in gametocytes and ookinetes. (E) The number of up- and down-regulated genes upon PfPHD1 PHD domain deletion are shown as the red and blue bars at four developmental stages. These altered genes were further classified as the putative target genes of AP2-LT and the remaining genes. The putative target genes of AP2-LT were significantly enriched in those down-regulated genes in the late stages of PfPHD1-ΔPHD::GFP parasites. R, ring; ET, early trophozoite; LT, late trophozoite; S, schizont. ***, P <0.001 (Fisher’s exact test). (PDF) [file ppat.1009351.s010.pdf]

S11 Fig

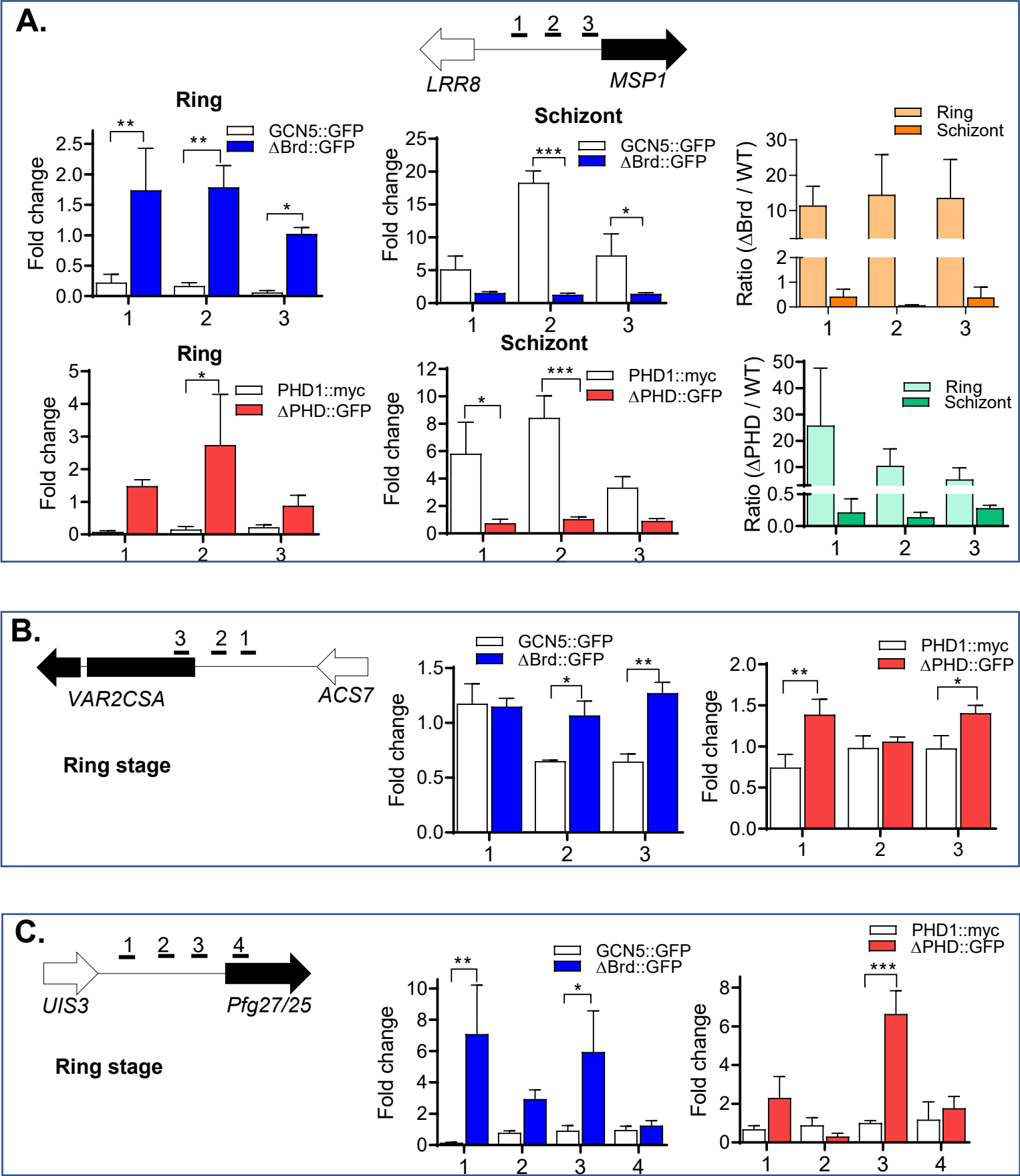

Supplement: S11 Fig — Genes selected for evaluation include MSP1 (A), VAR2CSA (B), and the sexual-stage gene Pfg27/25 (C). The enrichment was determined by chromatin-immunoprecipitation (ChIP) followed by qPCR using primer pairs marked as 1, 2, 3, or 4 located in the promoters of the respective genes. The fold change indicates the enrichment relative to constitutively expressed reference gene seryl-tRNA synthetase (PF3D7_0717700). (A) Upon domain deletion, PfGCN5-ΔBrD and PfPHD1-ΔPHD were depleted in the promoters of MSP1 at the schizont (central panels) but enriched at the ring stage (left panels). The right panels show the ratio of enrichments between domain deletions (PfGCN5-ΔBrD::GFP and PfPHD1-ΔPHD::GFP) and their wild-type parasites (PfGCN5::GFP and PfPHD1::myc). (B, C) At the ring stage, PfGCN5-ΔBrD and PfPHD1-ΔPHD were enriched at the promoters of var2csa (B) and Pfg27/25 (C). (*, **, and *** indicate P < 0.05, 0.01 and 0.001, respectively, Mann-Whitney U test). (PDF) [file ppat.1009351.s011.pdf]

S12 Fig.

A.

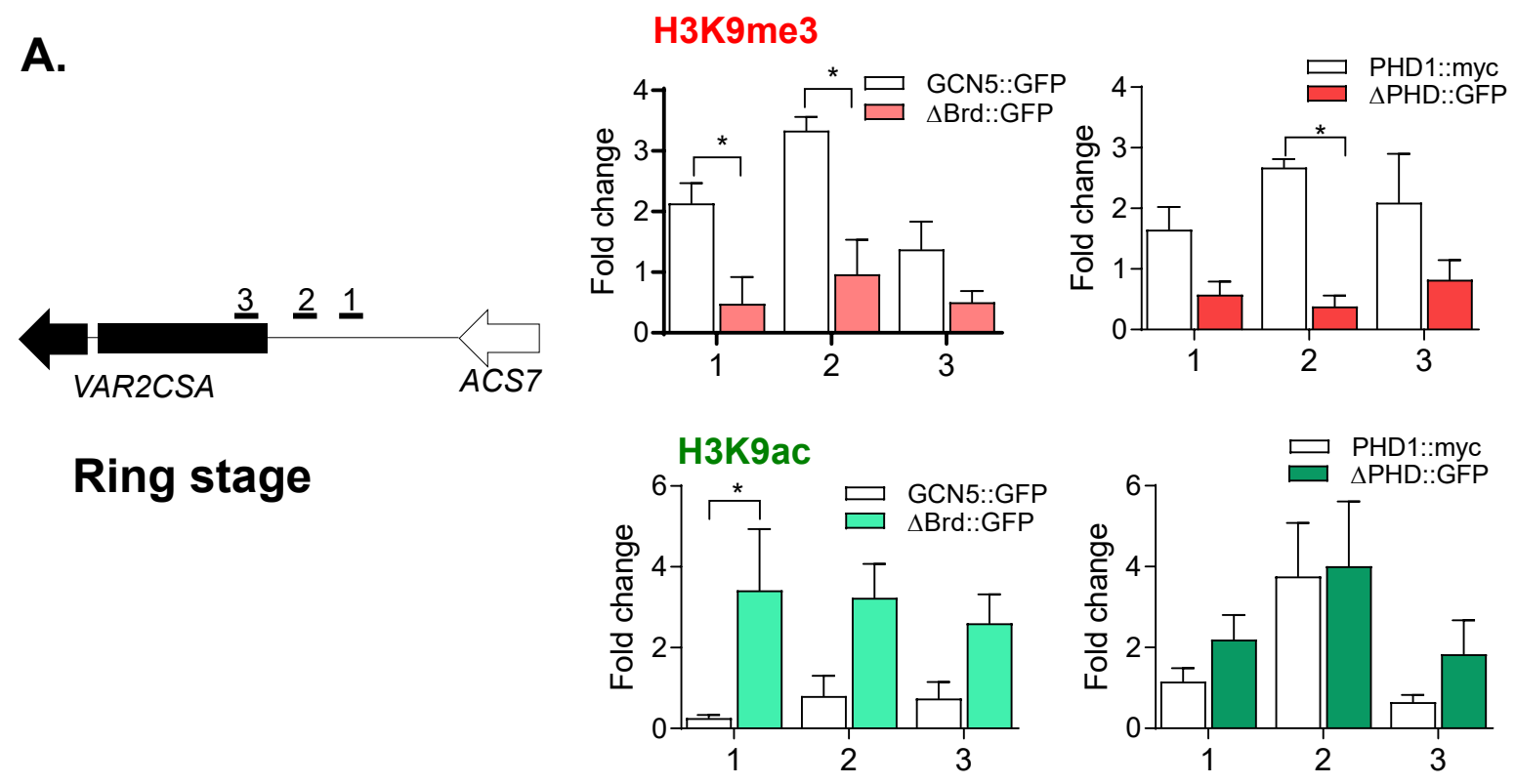

B.

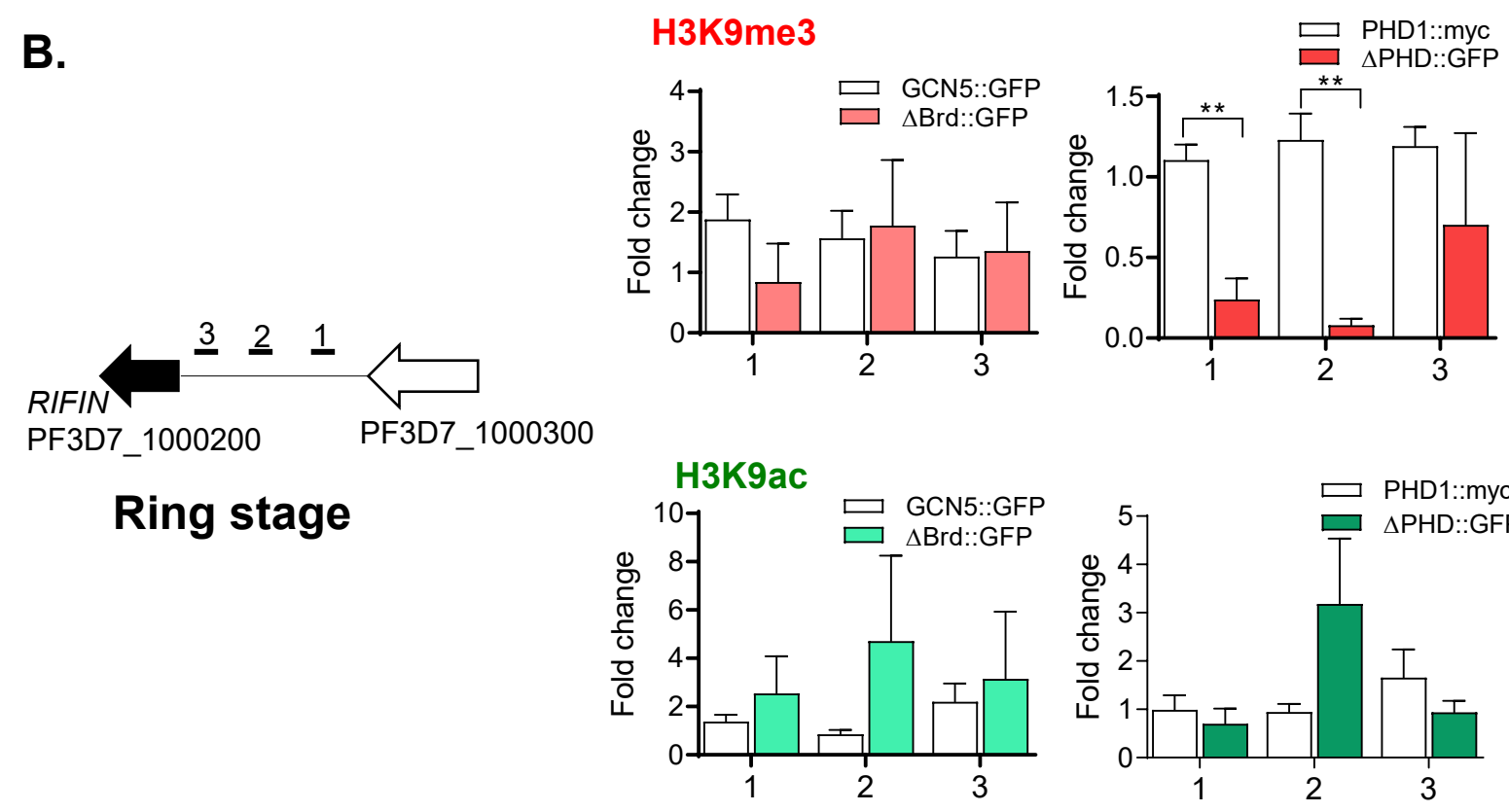

Supplement: S12 Fig — Selected HP1-controlled genes for evaluation include var2csa (A) and rifin (PF3D7_1000200) (B). Enrichment was determined at the ring stage by ChIP followed by qPCR using primer pairs marked as 1, 2, and 3 located in the promoters of the respective genes. The fold change indicates the enrichment relative to constitutively expressed reference gene seryl-tRNA synthetase (PF3D7_0717700). (A) H3K9me3 was depleted in the promoters of var2csa upon PfGCN5 BrD and PfPHD1 PHD deletion (upper panel) compared to the wild-type parasites (PfGCN5::GFP and PfPHD1::myc), whereas H3K9ac was enriched in both deletion mutants (lower panel). (B) Depletion of H3K9me3 and enrichment of H3K9ac were observed at certain positions of the rifin promoter upon BrD and PHD deletion, respectively. (PDF) [file ppat.1009351.s012.pdf]
