# Supplementary material for: A unique GCN5 histone acetyltransferase complex controls erythrocyte invasion and virulence in the malaria parasite Plasmodium falciparum
Source: PLoS Pathog. 2021 Aug 17;17(8):e1009351. doi: 10.1371/journal.ppat.1009351 (PMC8396726; doi:10.1371/journal.ppat.1009351)
Supplement: S2 Fig — (A) Sequence of four PHD domains in PfPHD1, C and H amino acid residues in the PHD domain are highlighted underneath the sequence. PHD-SF: PHD superfamily; ePHD: elongated PHD domain, PHD_TAF3: TAF3 type PHD domain. (B) Sequences of four PHD domains in PfPHD2. (C) Alignment of PfPHD1 PHD_TAF3 domain with other known authentic PHD domains which bind H3K4me3/2. The alignment shows the conserved Zinc-binding residues in light gray for Zinc 1 and dark gray for Zinc 2, and the two core β-strands in green. The residues involved in H3K4me3 recognition are labeled I through V (forming the aromatic cages) and the aromatic residues in the recognition cage are shadowed in green. MLL1: mixed-lineage leukemia-1; JARID1A: jumonji, AT-rich interactive domain 1A; PYGO: pygopus homolog 1; BPTF: bromodomain PHD finger transcription factor; PHF2: PHD finger protein 2; Yng1: yeast homolog of mammalian ING1; ING4: inhibitor of growth protein 4; TAF3: transcription initiation factor TFIID subunit 3. (PDF) [file ppat.1009351.s002.pdf]

S2 Fig

A. PfPHD1 PHD domains

```
PHD-SF 1540 LCNICNIGEDWEDNPIVFCDCCYTPMHSFCSGSKNI 1575
             C--C-------------C--C----H--C

ePHD 2383   CCICEFDAFYIGGGPIKRTKKKNEWCHIRCALISNCIINNKIEINNKEKTKYKCSLCRTNTTGIIKCNVPDCYKYYHISCATSSPKYLIELNDENKLILFCSNH 2487
            C--C-----------------------H--C------------------------C--C----------C----C----H--C-------------------C--H

ePHD 3200   CSYCPRLDGFLNCF-EDKGKKELLFGHPKCMEYVNTAYAKKNSVNDVKSGYNKRICSYCRIKHGVVITCSNTDCDVSFHISCGILLGCKMDNFFGRvdiyNPKKAYCFKH 3308
            C--C-----------------------H--C-------------------------C--C---------C----C----H--C------------------------C--H

PHD_TAF3 3794 YCPVCKSYYeelsdgspaDGLNWIGCDKCEKWYHWICCKYSVdnPPDIENDWYCNSC 3850
               C--C---------------------C--C----H-C------------------C--C
```

B. PfPHD2 PHD domains

```
PHD-SF 3971 NLMLKCSRCYMHVHKFCYISTKKSDEnilsniqnsasinsNEWLCQRC 4018
                 C--C----H--C----------------------------C--C

PHD-SF 4396 RCDVCLKNKGIFIKCHNMNCGKYIHPLCAYMCGLFIKCKNSNKKFlkfqNKIDYCF 4451
             C--C---------C----C----H--C--------------------------C-

PHD-SF 4577 CLVCFTSYKKSELVHCKYCNMCVHKNCYLVDSsyleyqfYRKKKNTCtLLVH 4604
            C--C-----------C--C----H--C--------------------C----H

PHD-SF 5394 NKNTCYICNLSYGYTQKCVENTCTHYFHISCARIHKLFFEFDYNFlkthpdstttdlskiPNSMIFCEDH 5463
                C--C---------C----C----H--C-------------------------------------C--H
```

C.

```
                      III          IV        V   II          I
MLL1     ------SGNFC--PLCDKCYDD-----DDYESKMMQCG---KC-DRWVHSKCENLSDEMYEILSNLPESVAYTCVN-CTERH------(1565-1628)
JARID1A  ---------SVCAAQNCQRPCK--------DKVDWVQCD--GGC-DEWFHQVCVGVSPEMAENA-------DYICIN-CA---------(1608-1659)
PYGO     ------PVYPC--GICTNEVN--------DDQDAILCE--ASC-QKWFHRICTGMTETAYGLLTAEAS-AVWGCDT-CMAD-------(337-396)
BPTF     ----DTKLY-C---ICKTPYD--------ESKFYIGCD---RC-QNWYHGRCVGILQSEAELI------DEYVCPQ-CQSTEDA----(2583-2639)
PHF2     --MATVPVY-C---VCRLPYD--------VTRFMIECD---AC-KDWFHGSCVGVEEEEAPDI------DIYHCPN-CEKTHGKSTLK(1-63)
Yng1     ----QEEVY-C---FCRNVSY----------GPMVACD-NPACPFEWFHYGCVGLKQ--APKG-------KWYCSKDCKEINAQRSKS(153-212)
ING4     -----EPTY-C---LCHQVSY----------GEMIGCD-NPDCSIEWFHFACVGLTT--KPRG-------KWFCPR-CSQE-------(195-245)
TAF3     RDEWGNQIWIC--PGCNKPDDG---------SPMIGCD---DC-DDWYHWPCVGIM-------AAPPEEMQWFCPK-CANKIKKDKKH(860-924)
PfPHD1   DTVDEEQIY-C--PVCKSYYEEISDGSPADGLNWIGCD---KC-EKWYHWICCKYSV----DNPPDIEN-DWYCNS-CLNS-------(3786-3853)
```

Loop 1 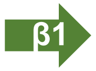 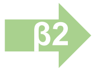 Loop 2
